# Post-Stroke Treatment with Neuromuscular Functional Electrostimulation of Antagonistic Muscles and Kinesiotherapy Evaluated with Electromyography and Clinical Studies in a Two-Month Follow-Up

**DOI:** 10.3390/ijerph19020964

**Published:** 2022-01-15

**Authors:** Juliusz Huber, Katarzyna Kaczmarek, Katarzyna Leszczyńska, Przemysław Daroszewski

**Affiliations:** 1 Department of Pathophysiology of Locomotor Organs, University of Medical Sciences, 28 Czerwca 1956 No 135/137, 60-545 Poznan, Poland; kat.leszczynska@gmail.com; 2 Neurology Ward, Pomeranian District Hospital, 75-581 Koszalin, Poland; kasia@przystan.pl; 3 Department of Organization and Management in Health Care, University of Medical Sciences, 60-545 Poznan, Poland; dyrektor@orsk.ump.edu.pl

**Keywords:** ischemic stroke, rehabilitation, kinesiotherapy, neuromuscular electrical stimulation, wrist and ankle antagonistic muscles, electromyography, Ashworth scale, Lovett scale

## Abstract

The aim of this study was to determine the sustained influence of personalized neuromuscular functional electrical stimulation (NMFES) combined with kinesiotherapy (mainly, proprioceptive neuromuscular facilitation (PNF)) on the activity of muscle motor units acting antagonistically at the wrist and the ankle in a large population of post-stroke patients. Clinical evaluations of spasticity (Ashworth scale), manual muscle testing (Lovett scale), and surface electromyography recordings at rest (rEMG) and during attempts of maximal muscle contraction (mcEMG) were performed three times in 120 post-stroke patients (T0: up to 7 days after the incidence; T1: after 21 days of treatment; T2: after 60 days of treatment). Patients (N = 120) were divided into two subgroups—60 patients received personalized NMFES and PNF treatment (NMFES+K), and the other 60 received only PNF (K). The NMFES+K therapy resulted in a decrease in spasticity and an increase in muscle strength of mainly flexor muscles, in comparison with the K group. A positive correlation between the increase of rEMG amplitudes and high Ashworth scale scores and a positive correlation between low amplitudes of mcEMG and low Lovett scale scores were found in the wrist flexors and calf muscles on the paretic side. Negative correlations were found between the rEMG and mcEMG amplitudes in the recordings. The five-grade alternate activity score of the antagonists’ actions improved in the NMFES+K group. These improvements in the results of controlled NMFES treatment combined with PNF in patients having experienced an ischemic stroke, in comparison to the use of kinesiotherapy alone, might justify the application of conjoined rehabilitation procedures based on neurophysiological approaches. Considering the results of clinical and neurophysiological studies, we suppose that NMFES of the antagonistic muscle groups acting at the wrist and the ankle may evoke its positive effects in post-stroke patients by the modulation of the activity more in the spinal motor centers, including the level of Ia inhibitory neurons, than only at the muscular level.

## 1. Introduction

Neuromuscular functional electrical stimulation (NMFES) of the paretic muscles, rather than functional electrical stimulation (FES) of the nerves, is the main supplementary method of physical therapy, commonly applied with pharmacological treatment and kinesiotherapy (conventional functional rehabilitation) in post-stroke patients [1,2,3]. The modern methods of rehabilitation, such as moving platforms, including treadmills with handrails, robot-assisted devices such as exoskeletons (including those electromyographically controlled), and robot-assisted gait-training devices in a virtual environment and in computer games (recently also using virtual reality devices), have rapidly developed in the treatment of post-stroke patients; however, the cost of their application is still high and not always available at rehabilitation centers [4,5]. Moreover, their efficiency in rehabilitation is still in clinical trials.

The therapeutic effect of electrical stimulation on muscle regeneration after its denervation from the level of spinal motoneuron has been evidenced, even in experimental studies on animals [6]. On the other hand, the justification of the clinical application of NMFES is limited to pathologies such as the consequences of spinal cord injury, stroke, and cerebral palsy involving the upper motoneuron. NMFES, including the one electromyographically triggered/controlled, has been shown to mainly improve the motor impairment of stroke survivors [7,8], probably by modifying the neural transmission in the synaptic contacts of the corticospinal tract with spinal motoneurons. NMES may be effective in neuromuscular rehabilitation by increasing motor unit recruitment, which is closely associated with an increase in muscle strength [9]. Available literature provides evidence on the specific effects of NMFES, depicting its important role in neuromodulation at the spinal and supraspinal levels in post-stroke patients [10]. Certain therapeutic applications of NMFES include facilitating upper and lower extremity motor relearning, reducing hemiplegic shoulder pain, strengthening muscles, and preventing muscle atrophy [7,11]. According to Stein et al. [12], NMFES combined with other intervention modalities can be considered as a treatment option that provides a reduction of spasticity and a range of motion improvement in post-stroke patients. It is possible that NMFES itself may evoke the transmission of neural impulses via biofeedback throughout the spinal reflex of the Ia inhibitory interneuron, which controls the alternate function of antagonistic muscles acting at the wrist and the ankle. The pathology of the coordination of these muscle groups is described as the most abnormal and problematic muscular dysfunction in post-stroke patients [13,14]. The spinal reflex of the reciprocal Ia inhibition constitutes a key segmental neuronal pathway for the coordination of antagonistic muscles in healthy people [15]. It is generally accepted that alpha motoneurons and Ia inhibitory interneurons are activated in parallel by the supraspinal centers facilitating a coordinated contraction of agonists and the relaxation of antagonists [16].

The review of the usage of NMFES in post-stroke patients revealed that it is delivered as a waveform of electrical current sequenced in pulsons and characterized by stimulus frequency, intensity, pulse width, and intervals between pulsons [12], and the moderate effectiveness of NMFES with various combinations of stimuli algorithms has been proven in the treatment of post-stroke patients, evaluated mainly with the clinical methods [13]. An optimal, suggested NMFES algorithm uses stimulation frequencies of about 15 Hz for upper extremity applications and about 20 Hz for lower extremity applications. The modified frequencies with proven efficiency have ranged from 10 to 50 Hz; sessions have usually been applied five times per week for 3–8 weeks. They have been applied with or without other rehabilitation modalities of treatment and have evoked the improvement of the results ascertained in the modified Ashworth scale, the muscle activity in the extensors of the wrist, increased dorsiflexor strength, decreased plantar flexor spasticity, scores of the Barthel Index, and the activities of daily living [17,18,19,20]. The objective and non-invasive neurophysiological evaluation of treatment, such as the electromyography recordings used in this study, has rarely been performed.

We hypothesize that, in the strategy of an NMFES application during treatment of post-stroke patients, attention should be drawn to the need for its polymodal (in relation to many muscles) application in closed (polysynaptically mediated) loops rather than in the open (monosynaptically transmitted) stimulation of a single muscle. It is obvious that the polymodal algorithm should include the NMFES applied rather in the alternate mode to the antagonistic muscles than to the synergistic ones. Few clinical applications of NMFES have involved a closed-loop control because of the numerous difficulties involved in its application if they are not performed in an alternative manner. Electrically evoked muscle contractions have presented the symptoms of fatigue; they could not be continuously sustained [21]. Moreover, none of the clinical trials on NMFES has included a precise neurophysiological evaluation of the patients’ current neuromuscular activity undertaken before the therapy. This seems crucial for the proper adjustment and safe electrotherapeutic procedures fulfilling the criteria of personalized therapy. Floeter et al. [22] hypothesized that learning a simple, alternating movement would produce changes in the spinal circuits that mediate reciprocal inhibition between the antagonistic muscles. In their study, the surface EMG recordings from the wrist flexor and extensor muscles showed reduced co-contraction during the acquisition of the task of flexion and extension.

There are a number of different approaches to physiotherapy treatment following stroke that are based on neurophysiological motor learning principles. Physiotherapists rarely base their treatment on a single approach; they mostly use a conjunction of components from a number of different methods. In terms of kinesiotherapy, the development of rehabilitation methods used in post-stroke patients was enriched in 1952 by Herman Kabat, who proposed a system of stretching exercises with the exact influence of the neurophysiological and neuroanatomical studies of Sir Charles Sherington, awarded with the Nobel prize in 1932. Taking into account Sherington’s findings described in four papers, *The Integrative Action of the Nervous System (1906)*, *Mammalian physiology (1919)*, *Reflex Activity of the Spinal Cord (1932)*, and *The Brain and Its Mechanism (1933)*, on the concept regarding the coordination of the motor center between the upper and lower extremities, Kabat together with his co-worker, a physiotherapist named Margaret Knott, evolved the system of stretching exercises aimed at generating the movement mechanisms contemporarily described as the reflex locomotion [23,24]. However, the effectiveness of proprioceptive neuromuscular facilitation (PNF) in the rehabilitation of post-stroke patients has only been documented in a few studies showing inconsistent benefits regarding decreased spasticity [25] and heterogeneous proposals of incorporation into functional training designed for stroke survivors [26]. PNF and Vojta’s stimulations were equally mentioned immediately after the most popular NDT Bobath method among kinesiotherapeutic procedures based on the neurophysiological approaches for the treatment of post-stroke patients [1,27]. Hypothetically, it can be supposed that the application of PNF may play a “facilitative” role for the other afferent impulses coming, for example, from electrotherapeutic stimulations, causing effects known in neurophysiology as the temporal and spatial summations of impulses to “the final common pathway”, i.e., the spinal motoneuron. Additionally, the mechanism of PNF is widely discussed; it is generally stressed that rehabilitation strategies that include activity-based therapy and targeted neuromodulation may use the anatomical connections between locomotor centers for the upper and lower extremities, originally described by Sherrington and Leslett as “the long propriospinal neurons” [28,29].

Lisiński et al. [14] and Kraft et al. [30] have similarly proved the effectiveness of the combination of stretching kinesiotherapy, including PNF and NMFES, in post-stroke patients, for the improvement of muscle activity in the upper and lower extremities. NMFES combined with PNF when applied to the muscles acting antagonistically at the wrist and ankle joints in post-stroke patients evoked better effects than PNF alone. However, in their studies, the treatment trial lasted only 20 days, and only a small group of patients was evaluated towards the effectiveness of the conjoined therapy (N = 24).

We undertook the presented study to determine the sustained positive results of such rehabilitation (during a s60-day observation) in a larger population of post-stroke patients (N = 120). Following the application of the modified NMFES algorithm, we hypothesized that the treatment may decrease the spasticity symptoms correlated with the EMG recording amplitude at rest and increase the muscle strength correlated with the EMG activity recorded during attempts of maximal muscle contraction. We also expected the improvement and maintenance of the proper alternate antagonistic muscle motor unit activity at the wrist and the ankle. This phenomenon may suggest the recovery of efferent neuronal signals from the motor cortex to the spinal centers following applied conjoined NMFES and PNF therapies in post-stroke patients.

## 2. Materials and Methods

### 2.1. Participants and Study Design

Clinical and neurophysiological studies were performed from December 2018 to April 2021 in the Neurology Ward in the Pomeranian District Hospital in Koszalin (Poland) and the Department of Pathophysiology of Locomotor Organs in University of Medical Sciences in Poznań (Poland).

**Figure 1:**
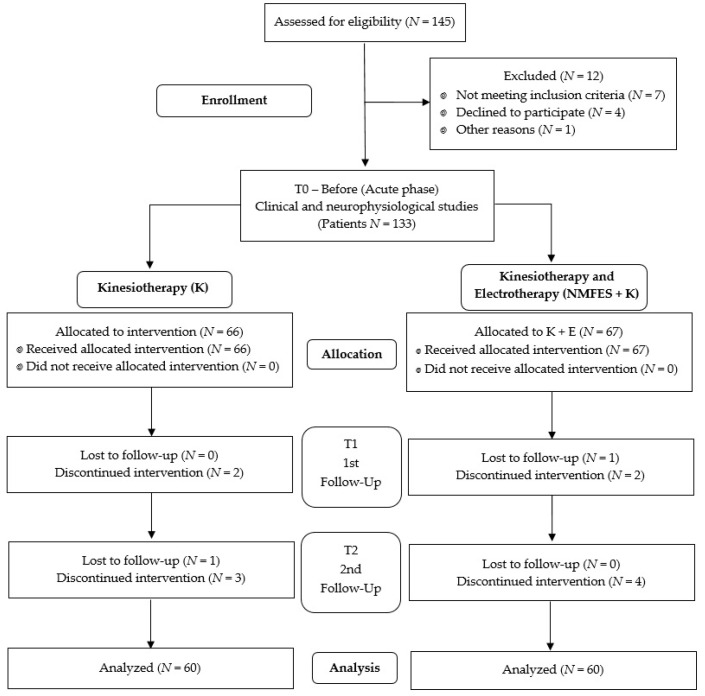
Flow chart of the study.

We recruited 145 people after ischemic stroke, clinically confirmed, and 60 healthy volunteers. Figure 1 summarizes all the stages of the study. Before the final analysis, we randomly decreased the number of patients (3 in the K group, and 4 in the NMFES+K group) (Figure 1). 

In the end, we used the data from 120 patients divided into two groups—one that has been treated only with kinesiotherapy (mainly PNF) performed and supervised by the team consisting of a physical and rehabilitation medicine physician and a physiotherapist, the K group, and another that received not only the same program of kinesiotherapy, but also electrotherapy, the NMFES+K group. Among these patients, in 65% we found symptoms of paresis in muscles of more upper than lower extremities on the right side. Both groups of patients and the healthy volunteers did not differ in demographic and anthropometric characteristics; all patients were treated with the same periods (Table 1).

The first incident of ischemic stroke had to be clinically confirmed on CT or MRI imaging performed in the acute phase of the incident (T0). None of the patients had been diagnosed with hemorrhagic stroke. Moreover, the main inclusion criteria for all patients were an age between 45 and 70 years, while the main contraindications for electrotherapy were epilepsy or previous consequences of ischemic stroke, severe disorders of the cardiovascular system, pregnancy, electronic implants such as pacemakers and cochlear implants, inflammatory diseases, proximal and distal neuropathy episodes in treatment (including COVID-19-related episodes), or myelopathies before the hospitalization. The patients who presented any of these contraindications for electrotherapy or strongly refused the application of the electrostimulation procedures were allocated to the K group. Therefore, we could not follow the rules of reporting randomized trials specified in CONSORT 2010 [31]; however, we followed the indications included in the STROBE statement for observational studies [32]. All patients understood the possibility of no benefits, agreed to participate in the study for no less than 3 months, and signed a written consent form.

**Figure 2:**
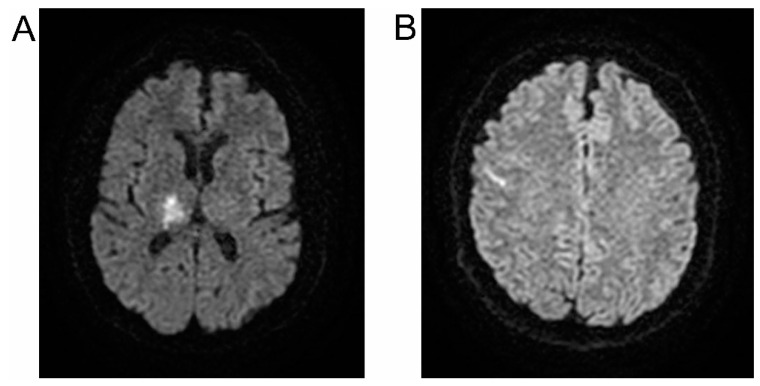
Examples of MRI pictures in DWI sequences presenting acute ischemic areas in patients from the NMFES+K group (**A**) and the K group (**B**).

Both the NMFES+K and K groups showed similar symptoms of ischemia in subcortical (55%) (Figure 2A) or frontoparietal (45%) (Figure 2B) areas on CT or MRI scans. The cross-sectional (coronal) area of ischemia averaged 276 mm^2^ ± 65mm^2^ in the K group and 297 mm^2^ ± 82mm^2^ in the NMFES+K group.

The study was conducted according to the guidelines of the Declaration of Helsinki and approved by the Bioethics Committee of the Medical University of Karol Marcinkowski in Poznań (Resolution 1279/18).

Outcomes were recorded following evaluations of both patients and healthy volunteers, with electromyography performed at rest and during attempts of maximal muscle contraction and with two clinical tests, the Ashworth Scale and the Lovett scale. Healthy volunteers were evaluated once to obtain reference values, while patients were evaluated three times in three different stages of the study: T0: at hospital ward up to 7 days after the incident; T1: after 21 days of treatment at the hospital ward; and T2: after 62 days of treatment in the rehabilitation center. The healthy volunteers were used as the control group to compare their results with both groups of patients.

### 2.2. Clinical Evaluation

The neurological status of all patients and the healthy volunteers was evaluated by the team of a physical and rehabilitation medicine physician and a physiotherapist. They used the Ashworth scale to measure spasticity, the clinical symptom of increased muscle tension not recorded in the patient’s history prior to the incident, and the Lovett scale to assess the muscle strength of the upper and lower extensors and flexors for both the wrist and the ankle. Although these scales are rather subjective, both are commonly used in neurology and rehabilitation to evaluate treatment and a patient’s treatment progress [33,34,35].

The Ashworth scale measures the resistance during passive soft-tissue stretching performed by an appraiser. It is a five-grade scale in which 0 stands for no increased muscle tension and 4 means no movement, i.e., the affected part is rigid in flexion or extension.

The Lovett scale consists of six grades that assess the different levels of muscle strength: 0 reflects no visible voluntary contraction of a muscle and 5 stands for normal muscle strength, i.e., a patient is able to perform exercises with resistance.

For comparison, both clinical tests were performed, once in the group of healthy volunteers and in three different stages of the study (T0, T1, and T2) in the groups of patients (K group and NMFES+K group). The results, including those obtained in the healthy people, are summarized in Table 2. 

In our opinion, the normative values of clinical and neurophysiological studies in healthy people need to be obtained every five years due to the changes caused by increasingly common sedentary lifestyles, which may influence the function of the musculoskeletal system. Moreover, we would like to compare the results obtained from healthy people with those of post-stroke patients to highlight the differences.

### 2.3. Kinesiotherapy

On about the third day after admission in the neurological ward, the patients’ general health status was stable enough to begin the physiotherapeutic treatment. During the first 10 days of staying in the hospital, physiotherapists taught patients how to hold an upright position, sit down, change positions, and perform global movements necessary for everyday life (e.g., changing position in bed and changing from a wheelchair to a chair). Afterwards, patients were taught proper locomotion, including walking with handrails and walking with orthopedic equipment (e.g., a walker or a walking stick).

**Figure 3:**
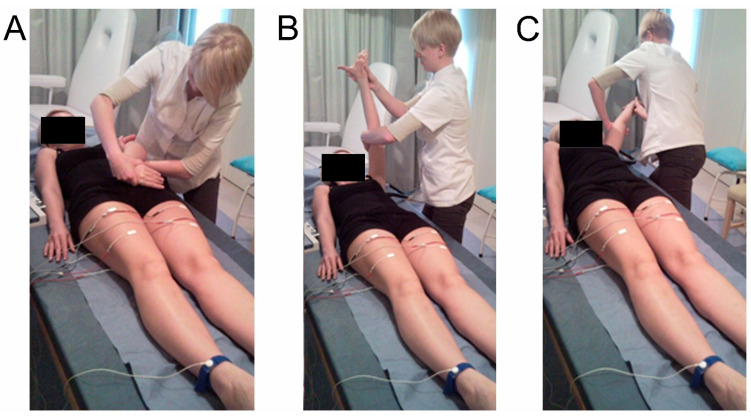
Examples of the PNF pattern ((**A**)—flexion, (**B**)—abduction, and (**C**—external rotation) applied to the upper extremity of post-stroke patients. Control electromyographical recordings from the rectus femoris muscles bilaterally detected the simultaneous muscle motor unit activity predominantly on the contralateral side, proving the properness of the applied therapy and the transmission of neuronal signals from the cervical to lumbosacral spinal neuromeres, probably via the fibers of the long crossed propriospinal neurons.

In general, the same kinesiotherapy program, carried out with the same intensity and based mainly on PNF, was applied to both groups of patients. Thus, one group of patients received only kinesiotherapy (K group), while the other received kinesiotherapy and electrotherapy (NMFES+K group). Physiotherapists administered an exercise program based on the PNF patterns of flexion, abduction, and external rotation as well as extension, abduction, and internal rotation for the paralyzed upper and lower extremities, respectively (Figure 3) [23,36]. Other parts of the training were individualized; therapists adapted them to the psychophysical state of the patient. The additional training mainly included passive, supportive, and active exercises on the paretic side, exercises reducing the spasticity symptom (PIR: post-isometric relaxation treatments), and stretching exercises, which stimulate proprioceptors.

Patients were clinically examined and neurophysiologically evaluated at T0 in the acute phase up to 7 days of staying in the neurological rehabilitation ward, and subsequently in the subacute phase in the rehabilitation center belonging to the neurology ward in the hospital (at T1, after 21 days of treatment, and at T2, after 62–63 days of treatment). Both groups of patients received treatment every day except Saturdays and Sundays (5 days a week), and one session a day lasted about 3 h. In addition, “warming therapy” was provided to all patients.

### 2.4. Neuromuscular Functional Electrical Stimulation (NMFES) Algorithm Based on Neurophysiological Evaluation

The neuromuscular functional electrical stimulation (NMFES) of antagonistic muscles acting at the wrist and the ankle was applied only to the NMFES+K group. The development of the electrotherapy principles and the details of personally adjusting the stimuli algorithm have been described elsewhere [14,37]. All details regarding the stimulation parameters applied to the NMFES+K group are presented in Table 1.

**Figure 4:**
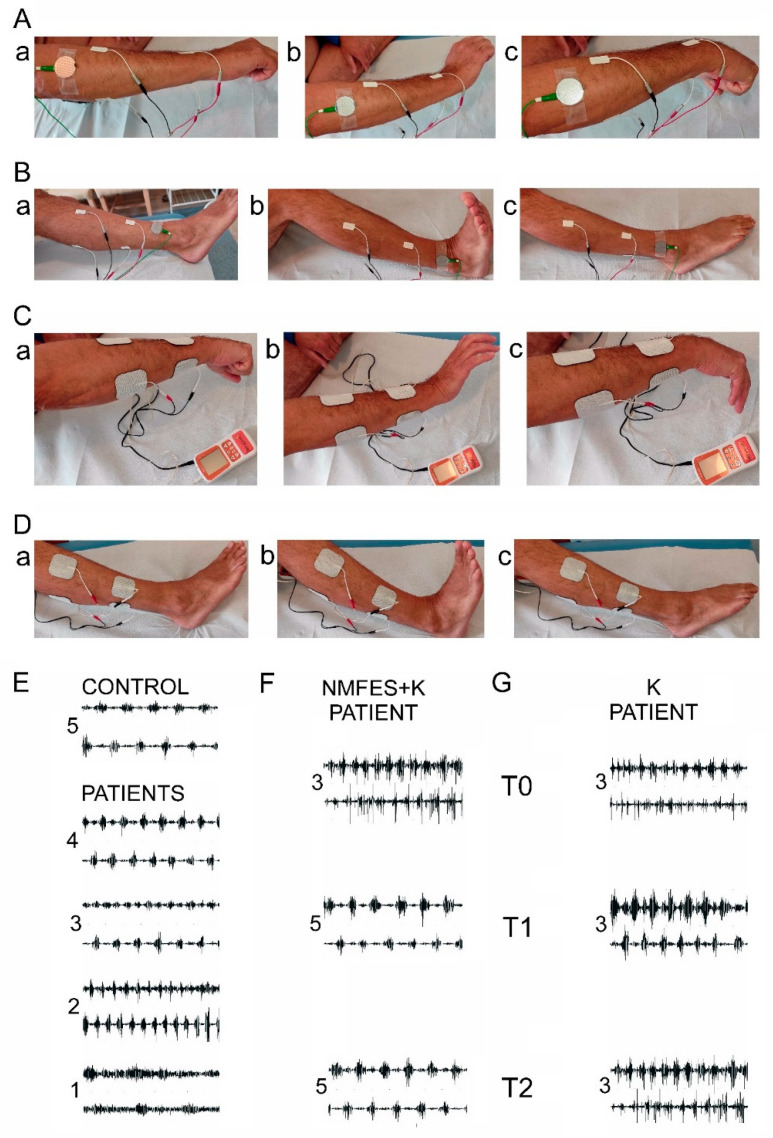
(**A**,**B**): Distribution of recording electrodes on the surface of the skin over the extensor and flexor muscle groups at the wrist (**A**) and the ankle (**B**) in the neutral position at rest (**a**), during attempts of maximal contraction of the extensors (**b**), and during attempts of maximal contraction of the flexors (**c**). (**C**,**D**): Location of the stimulating surface electrodes used for NMFES of the antagonistic muscle groups at the wrist (**C**) and the ankle (**D**) at the neutral position (**a**), evoking the extension (**b**), and evoking the flexion (**c**). Examples of EMG recording patterns showing the alternate function of the antagonistic muscle groups acting at the wrist (**E**) under normal conditions (control, 5) and in patients with varying degrees of severity (patients, 4–1) (E5: full alternate, normal activity; E4: slightly disturbed alternate activity; E3: moderate disturbed alternate activity; E2: highly disturbed alternate activity; E1: no alternate activity). Examples of EMG recorded in one of the patients from the NMFES+K group (**F**) and the K group (**G**) before treatment (T0) and in two observation periods (T1: after 21 days of treatment; T2: after 60 days of treatment). Note the improvement of the antagonistic muscle groups acting at the wrist only in the patient from the NMFES+K group.

A mobile, personal, four-channel device (NeuroTrac^®^ Sports XL, Verity Medical Ltd., Hampshire, UK) was used for the stimulation of the antagonistic muscle groups acting at the wrist (Figure 4(Ca)–(Cc)) and the ankle (Figure 4(Da)–(Dc)). 

The locations of the stimulating electrodes were the same as the motor points over the extensor carpi muscle group and the flexor carpi muscle group (at the wrist; Figure 4(Aa)–(Ac)) and the motor points over the tibialis anterior muscle versus the calf muscle group (the medial and lateral gastrocnemius muscles and the soleus muscle at the ankle; Figure 4(Ba)–(Bc)), where sEMG recordings were also performed. Two pairs of self-adhesive surface electrodes (AxelgaardUltrastim Wire Neurostimulation Electrodes with MultiStick Gel, 5 cm × 5 cm, Axelgaard Manufacturing Co. Ltd., Lystrup, Denmark) were placed over the skin according to the anatomical location of the muscle. The anode was placed on the muscle belly; the cathode was placed on the distal tendon of the muscle. NMFES was applied polymodally in an alternative mode, which means that the stimulation device released, via two pairs of bipolar surface electrodes, trains of electrical stimuli exciting the first flexor and then the extensor muscle groups at the wrist and the ankle (Figure 4C,D). According to the neurophysiological terminology, we used electrical bipolar, rectangular pulses, with subsequent upper and lower inflexions, which were negative and positive.

We used sEMG recordings from the same muscle leads mentioned above (Figure 4(Aa)–(Ac),(Ba)–(Bc)); they were also used for the purpose of electroneurography (ENG) recorded at T0. During the ENG recordings, maximally evoked M-wave amplitudes following electrical excitation of motor fibers in ulnar and peroneal nerves were analyzed to determine the stimulus strength, in milliamperes, necessary to evoke them. This neurophysiological evaluation allowed for the creation of the individually adjusted electrostimulation algorithm applied to the patients from the NMFES+K group (Table 1).

In terms of the consequent algorithm parameters, the frequency of stimuli in one train delivered from the electrodes depended on the sEMG frequency parameter recorded during an attempt of a maximal muscle contraction (35–70 Hz, 48.6 Hz on average), the interval between the bursts of pulsons was from 2 to 5 s (4.3 s on average), the single stimulus duration was calculated from the repetitive measurements of the successive duration of single muscle motor action potentials in sEMG recordings (14.1 ms on average), and the stimulus intensity set up for the muscles of the upper and lower extremities was 26.8 mA on average. The latter parameter was calculated from the stimulus strength applied to the ulnar and peroneal nerves to evoke the maximal amplitude of an M-wave response. The team consisting of a physical and rehabilitation medicine physician and a physiotherapist set up and supervised all parameters. The stimulating device, given to a patient and personalized, was blocked after programming the stimuli algorithm. This prevented unplanned changes applied by the patients up to T1, when the algorithm could be verified and modified. The participants could change only the stimulus strength parameter. They were instructed to increase the stimulus strength during the single stimulation session so that the visible contraction of the stimulated muscles could be observed and achieved without intrusive pain. The duration of one session depended on the severity of neurogenic changes ascertained in sEMG recordings from 15 to 20 min (19.1 min on average). NMFES sessions were performed five times a week for a period of no less than 2 months. Data in Table 1 indicate that patients were treated from 50 to 72 days, 62 ± 6 days on average. The memory of the device includes the settings and the read-outs—both storages were used to verify the therapy course at T1 and T2. Other principles of the neurophysiological methodology of EMG and ENG recordings, including the measurement outcomes and their interpretation for clinical practice, are presented in detail in other papers [38,39].

No electrotherapy treatments other than NMFES described in the study were performed.

### 2.5. Data Analysis

Statistica software, version 13.1 (StatSoft, Kraków, Poland), was used for the data analysis. Descriptive statistics included mean and median values, standard deviations (SDs), and minimum (min) and maximum (max) values for measurable variables. Shapiro–Wilk tests and Levene’s tests were used to determining the normality distribution and homogeneity of variances. The Student’s *t*-test and Mann–Whitney test were used to compare the mean and median values of parameters from neurophysiological and clinical studies in 120 post-stroke patients. A significant statistical difference was determined at *p* ≤ 0.05 during the comparison. Before the study was completed, preliminary analysis revealed the required sample size using the primary outcome variables from mcEMG recordings before and after treatment with a power of 80% and a significance level of 0.05 (two-tailed). The data from the first 20 subjects were used to calculate the mean and standard deviation (SD). The sample-size software estimated that the minimum number of subjects was 45.

The non-parametrical Spearman’s rank correlation coefficient (r_s_) was used to demonstrate correlations between Ashworth’s or Lovett’s scale scores and rEMG or mcEMG amplitude measurement results, respectively. A *p* ≤ 0.05 significance level was assumed as statistically significant for rank correlation.

## 3. Results

Side effects were not reported by any of the participants. Table 1 presents the data proving that the difference between the expected and detected durations of applied electrostimulations was small, which means the patients followed the therapy protocol of NMFES instructed at T0. All the stimulation parameters were personally adjusted based on the preliminary neurophysiological evaluation of every patient with EMG and ENG studies. The details of the electrotherapy algorithm are presented in Table 1. The most important parameters for the comparison with the other applications of NMFES available in the literature, which are presented in the Introduction Section, were the frequency of train at 48.6 Hz on average and the stimulus strength intensity of about 26.5 mA on average; they were read out from the devices after the trial was completed at T2.

Both groups of patients presented similar symptoms of spasticity in the flexor muscle group and a low strength in both flexors and extensors in the upper and lower extremities (Ashworth score: 3; Lovett scale score: 2–3; see Table 2). Results gathered in Table 2 also indicate a significant improvement in the Ashworth scale results in flexor group muscles, more than extensor group muscles, both in the upper and lower extremities, but only in patients from the NMFEs+K group and not in the patients treated with kinesiotherapy alone. Lovett scale scores improved significantly only in the NMFES+K group. It should be highlighted that there are no statistically significant differences among the healthy people and the NMFES+K group only in the Ashworth scale at T2, after 60 days of treatment, while the differences remained significant for the K group in both the Ashworth and Lovett scale scores, indicating the lack of remarkable improvement.

Data in Table 3 indicate that the electromyography amplitudes recorded in both groups of patients differed significantly at T0 in relation to the normal parameters recorded in the healthy people. In general, the parameters of the rEMG recordings in patients of both groups were greater than 25 µV on average, which indicates increased muscle tension (clinically a spasticity) [38,39], manifested more in the flexors than the extensors. In contrast, mcEMG recordings of the patients were significantly lower in amplitudes when compared to the healthy subjects; similarly, the pathological changes were more significant in the flexors.

In general, the same recordings performed at T2 indicate the significant improvement of the amplitude parameter (decreased rEMG and increased mcEMG that were not statistically different from the healthy people) in the NMFES+K group patients. The K group patients’ results were not characterized with a similar improvement; their results at T2 remained significantly worse. Note that a preliminary improvement in the patients from the NMFES+K group was already observed at T1.

Data in the bottom of Table 3 and the examples of the EMG recordings of the alternate muscle contraction in Figure 4F suggest that the index of the antagonistic muscle action both at the wrist and the ankle improved in the patients from the NMFES+K group more than in the K group (Figure 4G). Both groups of patients before the therapy showed an index of 3; after the therapy, it was found to be 5, but only in the NMFES+K group. This is comparable to the pattern recorded in the healthy volunteers (Figure 4A).

Correlation studies of the examined parameters at T0 were found to be significant at *p* ≤ 0.05 for the wrist flexor and the calf muscle recordings in both groups of patients. Data in Table 4 present positive correlations between the increased rEMG amplitudes values and high Ashworth scale scores. A significant positive correlation was also detected between the decreased amplitude parameter in mcEMG recordings and the low Lovett scale scores. Negative correlations were found between the rEMG and mcEMG amplitudes in recordings. The correlations were more significant at T0 than at T2 and in patients of the NMFES+K than those of the K group.

## 4. Discussion

This study was performed on a population of post-stroke patients divided into two similar groups, taking into account their health status before the treatment was applied. We present evidence for an improvement in antagonistic muscle activity (more flexors than extensors) following combined NMFES+K therapy over activity following PNF kinesiotherapy alone. Thus, we verified the hypothesis that physiotherapy intervention using conjoined components from different neurophysiological approaches of treatment is significantly more effective than kinesiotherapy alone. We fulfilled the proposal of Pollock et al. [40] and Hong et al. [41], who, in their meta-analysis, recommended performing research that should concentrate on studies determining the effectiveness of clearly described individual techniques and task-specific treatments applied separately or as combinations. Our results also prove, with neurophysiological methods, the moderate efficiency of PNF therapy as a single method of treatment, which is in agreement with Anas et al. [25] and Guiu-Tula et al. [26], who, with the methods of clinical evaluation, obtained heterogeneous evidence of the benefits of PNF intervention.

Our study focused on the application of NMFES, in a closed loop, involving the activation of the spinal centers, following the polymodal, but not unimodal, stimulation of muscles. Such an approach is scarcely presented in the literature. Exceptionally, Floeter et al. [22] proved that learning a simple, alternating movement would produce changes in the spinal circuits that mediate reciprocal inhibition between antagonist muscles. In their studies, surface electromyography recordings from the wrist flexor and extensor muscles showed reduced co-contraction during the acquisition of flexion and extension tasks—similar to our study. Most of the studies on an NMFES application in post-stroke patients are applied to a single muscle; moreover, the results are usually evaluated with clinical, often subjective tests [11,12], and not with objective neurophysiology methods such as rEMG and mcEMG. However, they similarly described the effectiveness of NMFES up to 8 weeks following its application, in terms of decreased spasticity and increased motor performance, when the frequency of the applied stimulation ranged, as in our study, from 35 to 50 Hz [17,19,20,42,43].

**Figure 5:**
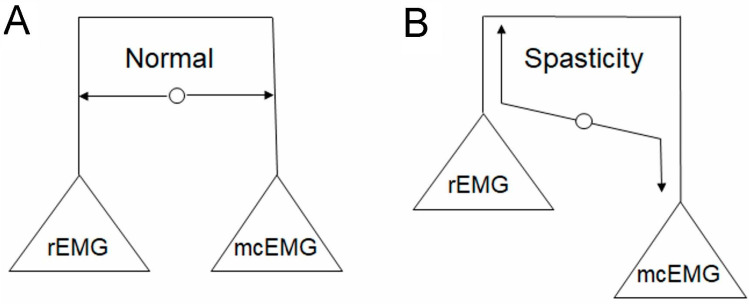
Illustration of the relationship between the amplitude parameters of electromyography recordings at rest (rEMG) and during attempts of maximal muscle contraction (mcEMG) in a healthy subject (**A**) and a post-stroke patient before therapy (**B**). Note the abnormal increase if the muscle tension at rest leads to a decrease in muscle motor unit activity during voluntary movement.

The mechanism by which NMFES recovers the lack of descending control of spinal circuits in post-stroke patients is explained by the increase in the presynaptic inhibition of muscle spindle reflex activity [43], the influence of the cortical neuroplasticity via the long-loop biofeedback control [44], or the change at the level of the muscle by the increased efficiency of the muscle motor units themselves [45]. The results of our study may indirectly indicate the effectiveness of NMFES and PNF applied together based on the results of rEMG and mcEMG, revealing the functional reorganization in the neuronal centers in the spinal cord and at the supraspinal level. Such a conclusion can be drawn from the correlations found in this study between the clinical scales of spasticity, the muscle strength evaluation, and the resting muscle activity (rEMG) and the activity during attempts of maximal muscle contraction (mcEMG), and such findings have never been presented before with clinical neurophysiological methods (Figure 5).

Balance can be observed between rEMG and mcEMG amplitudes recorded in a normal state in healthy subjects (Figure 5A), when the transmission of the nerve impulses from the neurons of the ventral horn to the effector is at 5 Hz with simultaneously proper inhibitory and excitatory actions that are mediated polysynaptically to the motoneuron from, mainly, the fibers of the corticospinal tract. One of the crucial spinal systems responsible for such a relationship is the one in which the Ia inhibitory interneuron regulates the action of antagonistic muscles acting at the same joint [46]. In cases of disturbances of neural activity at the cortical level, like in post-stroke patients, the frequency of discharges from the level of the lower motoneuron to the effector increases, which evokes increased muscle tension, and consequently decreases the ability of muscle motor units to perform proper muscle contraction [47] (Figure 5B). The spatial facilitation of afferent influences coming from such sources as NMFES and PNF applied by a physiotherapist, especially in the alternative mode to the flexors and extensor acting at the same joint, may compensate the disturbed efferent actions of neuronal signals of a supraspinal origin in post-stroke patients. The patients from the NMFES+K group nearly presented the proper alternate antagonistic muscle motor unit activity at the wrist and the ankle (index: 5; Table 3 and Figure 4F) after 60 days of therapy. This might contribute to the recovery of the proper balance between the amplitudes in the rEMG and mcEMG recordings, which, in the patients from the NMFES+K group, were closer to normal at T2 (Table 3). The facilitating role of PNF treatment for the recovery of motor function in post-stroke patients might address not only the cervical and lumbosacral spinal levels within “central pattern generators” [36], but also the neuroplasticity processes at the cortical areas both ipsi- and contralaterally to the ischemic changes [48], which are known to be therapeutically influenced by PNF procedures, according to Sharman et al. [36]. Recently, Piscitelli et al. [49] provided the evidence on the role of corticospinal excitability and the tonic stretch–reflex thresholds modulation. This ability is impaired in stroke and contributes to sensorimotor impairments such as spasticity.

Further evidence on the greater efficacy of therapy demonstrated in the NMFES+K patients may involve the level of the muscle itself, considering the influence of electrical stimulation, which spreads within the intramuscular branch in orthodromic and antidromic directions [10]. Stimuli depolarizing the spinal motoneurons on the reflex (within the afferent part) and the recurrent way (in the efferent part), together with protective actions of electrical charges within motor axons, may play an additional role in sustaining or increasing the motor units’ contractile properties. However, such positive effects of therapy are possible only when the algorithm of applied stimuli is personally adjusted to the current functional state of the muscle motor unit activity in a certain post-stroke patient. These prerequisites were fulfilled in the present project by examining patients from the NMFES+K group at T0 with the use of rEMG and mcEMG as well as ENG tests.

In this paper, we confirm the results of previous short-observation studies on the effectiveness of NMFES conjoined with PNF kinesiotherapy in post-stroke patients [14,30] as well as the findings of Sahin et al. [50], who, using a frequency of 100 Hz for wrist extensor stimulation, also proved that NMFES was more effective than PNF stretching of wrist extensor muscles alone in reducing spasticity.

The limitation of this study might be the joint stiffness following the long-lasting closed-loop electrical stimulation reported by Bó et al. [51]; however, none of the patients stimulated in our project complained of this symptom. Another study limitation could be that the observation duration lasted only two months. However, we focused on applying the same form of treatment in two groups of patients, which was directly supervised by the same team of specialists, making the assessors not blind. This is possible only if patients are treated in the same rehabilitation center belonging to the neurology ward in the public hospital. Unfortunately, treatment in hospitals covered by insurance usually lasts no longer than two months. After this period of time, patients are treated in different outpatient clinics, which may influence treatment outcomes. Due to contraindications as well as a lack of agreement for electrostimulation procedures from some of the K group patients, this study was not randomized.

The results of our study prove the sustained effects of NMFES+K treatment, possibly due to a unified treatment regime. There is a discrepancy between the mean age of the healthy subjects and that of the two groups of post-stroke patients, presented in Table 1. We used healthy subjects only to compare how post-stroke patients’ results differ prior to and after the therapy.

## 5. Conclusions

Following the application of personalized, closed-loop alternate NMFES conjoined with PNF treatment in post-stroke patients over 60 days of observation, we found its positive effects by means of a decrease in spasticity symptoms correlated with the EMG recording amplitude at rest and an increase in muscle strength correlated with the EMG activity recorded during attempts of maximal muscle contraction on the paretic side. The patients who received the same form of the treatment nearly presented the proper alternate antagonistic muscle motor unit activity at the wrist and the ankle after 60 days of therapy. The results of our study imply the moderate, but indispensable facilitating role of PNF procedures in the recovery of motor control in post-stroke patients. The better results of the controlled NMFES treatment combined with the PNF method in comparison with the use of kinesiotherapy alone justify the application of conjoined rehabilitation procedures based on neurophysiological approaches and that it should be more often implemented in stroke survivor treatment programs. Considering the results of both clinical and neurophysiological studies, we postulate that NMFES of antagonistic muscle groups acting at the wrist and the ankle may evoke positive effects in post-stroke patients by the modulation of activity more in the spinal motor centers, including the level of Ia inhibitory neurons, than only at the muscular level.

## Figures and Tables

**Table 1:** Characteristics of the subjects and the summary of applied electrotherapy parameters.

Study Group Variable	Healthy Volunteers (Control), N = 60, 41♀, 19♂		NMFES+K Group Patients, N = 60, 44♀, 16♂		K Group Patients, N = 60, 45♀, 15♂	
	Mean ± SD	Min–Max	Mean ± SD	Min–Max	Mean ± SD	Min–Max
Age	48.6 ± 4.3	30–52	62 ± 6.1	47–70	65 ± 5.2	56–70
Height (cm)	166.0 ± 4.8	161–180	163 ± 10.3	148–178	167 ± 7.2	157–180
Weight (kg)	75.3 ± 9.5	52–81	72 ± 11.1	55–95	74 ± 11.4	52–98
Observation time (days)	NA	NA	62 ± 6	50–72	63 ± 6	50–72
Expected stimulation (hours)	NA	NA	19.2 ± 2.1	15–23	NA	NA
Detected stimulation (hours)	NA	NA	18.4 ± 4.3	16–24	NA	NA
Train stimulation frequency (Hz)	NA	NA	48.6 ± 6.1	35–70	NA	NA
Single stimulus duration (ms)	NA	NA	14.1 ± 15.2	12.5–17.5	NA	NA
Train duration (s)	NA	NA	4.1 ± 1.7	3–6	NA	NA
Interval between trains (s)	NA	NA	4.3 ± 1.2	2–5	NA	NA
Session duration (mins)	NA	NA	19.1 ± 2.2	15–20	NA	NA
Applied stimulus strength (mA) Upper extremity muscles -flexors -extensors	NA	NA	25.9 ± 3.1 26.2 ± 3.0	27–33 21–35	NA	NA
Applied stimulus strength (mA) Lower extremity muscles -flexors -extensors	NA	NA	25.4 ± 3.1 28.2 ± 3.3	21–37 23–32	NA	NA

NA: Not applicable.

**Table 2:** Comparison of results from clinical studies recorded in two groups of patients and healthy subjects. Results refer to recordings performed in patients on a paretic side identified in the preliminary clinical examinations. When comparing the results of the Ashworth and Lovett scale tests, they were cumulatively averaged for the examination of the extensor and flexor muscles of the upper and lower extremities. Ranges and median values are presented.

Test or Parameter	Healthy Volunteers N = 60	T0 Acute Phase (Up to 7 Days after Incident)		T1 Subacute Phase (After 21 Days of Treatment)		T2 (After 62 Days of Rehabilitation Center Treatment)		*p* Patients T0 vs. T2 Before–After	*p* Healthy vs. Patients T0 Before	*p* Healthy vs. Patients T2 After
		Group NMFES+K Patients N = 60	Group K Patients N = 60	Group NMFES+K Patients N = 60	Group K Patients N = 60	Group NMFES+K Patients N = 60	Group K Patients N = 60			
Ashworth scale (+4–1)								NMFES+K	NMFES+K	NMFES+K NS
-upper flexors	[1–1] 1	[1–2] 1	[1–2] 1	[1–1] 1	[1–2] 1	[1–1] 1	[1–2] 1	***p* = 0.04**	***p* = 0.03**	
-upper flexors	[1–1] 1	[2–4] 3	[2–4] 3	[1–3] 2	[2–4] 3	[1–1] 1	[1–3] 2	K *p* = 0.05	K ***p* = 0.03**	K *p* = 0.05
								NMFES+K	NMFES+K	NMFES+K NS
-lower extensors	[1–1] 1	[1–2] 1	[1–2] 1	[1–1] 1	[1–2] 1	[1–1] 1	[1–2] 1	***p* = 0.04**	***p* = 0.03**	
-lower flexors	[1–1] 1	[2–4] 3	[1–4] 3	[1–4] 2	[2–4] 3	[1–2] 1	[1–4] 3	K NS	K ***p* = 0.03**	K ***p* = 0.03**
Lovett										
scale (0–5)								NMFES+K	NMFES+K	NMFES+K
-upper extensors	5	[2–4] 3	[2–4] 3	[3–5] 4	[2–4] 3	[3–5] 4	[3–4] 3	***p* = 0.04**	***p* = 0.02**	*p* = 0.05
-upper flexors	5	[1–3] 2	[1–3] 2	[2–4] 3	[1–3] 2	[3–5] 4	[2–4] 3	K *p* = 0.05	K ***p* = 0.02**	K ***p* = 0.04**
								NMFES+K	NMFES+K	NMFES+K
-lower extensors	5	[2–4] 3	[2–4] 4	[3–5] 3	[2–4] 4	[2–5] 4	[2–4] 4	***p* = 0.03**	***p* = 0.03**	*p* = 0.05
-lower flexors	5	[1–4] 3	[2–4] 3	[2–5] 4	[2–4] 3	[3–5] 4	[2–4] 3		K ***p* = 0.04**	K ***p* = 0.04**

Abbreviations: NS: not significant.

**Table 3:** Comparison of results from electromyographical recordings in the two groups of patients and the healthy subjects.

Muscle Group	Healthy Volunteers N = 60	T0 Acute Phase (Up to 7 Days after Incident)		T1 Subacute Phase (After 2–3 Weeks of Treatment)		T2 (After 2 Months of Rehabilitation Center Treatment)		p Patients T0 vs. T2 Before–After	p Healthy vs. Patients T0 Before	p Healthy vs. Patients T2 After
		Group NMFES+K Patients N = 60	Group K Patients N = 60	Group NMFES+K Patients N = 60	Group K Patients N = 60	Group NMFES+K Patients N = 60	Group K Patients N = 60			
rEMG (Amplitude at Rest in µV)										
-wrist extensors muscles	25 ± 6	37 ± 6	32 ± 4	35 ± 2	35 ± 3	28 ± 4	36 ± 5	NMFES+K ***p* = 0.03** K ***p* = 0.04**	NMFES+K ***p* = 0.02** K ***p* = 0.03**	NMFES+K NS K ***p* = 0.03**
-wrist flexor muscles	20 ± 4	64 ± 4	70 ± 3	55 ± 2	72 ± 3	43 ± 8	68 ± 5	NMFES+K ***p* = 0.009** K NS	NMFES+K ***p* = 0.008** K ***p* = 0.009**	NMFES+K ***p* = 0.03** K ***p* = 0.008**
-anterior tibial muscle	19 ± 3	40 ± 2	46 ± 3	39 ± 3	41 ± 4	20 ± 4	45 ± 4	NMFES+K ***p* = 0.008** K NS	NMFES+K ***p* = 0.008** K ***p* = 0.009**	NMFES+K NS K ***p* = 0.02**
-calf muscles	21 ± 2	95 ± 4	105 ± 7	22 ± 3	96 ± 1	23 ± 2	100 ± 6	NMFES+K ***p* = 0.009** K NS	NMFES+K ***p* = 0.009** K ***p* = 0.009**	NMFES+K NS K ***p* = 0.009**
mcEMG (Amplitude during Maximal Contraction in µV)										
-wrist extensors muscles	1385 ± 226	926 ± 129	919 ± 107	980 ± 222	801 ± 186	1290 ± 102	819 ± 100	NMFES+K ***p* = 0.04** K NS	NMFES+K ***p* = 0.03** K ***p* = 0.03**	NMFES+K NS K ***p* = 0.03**
-wrist flexor muscles	1622 ± 428	821 ± 232	795 ± 121	1325 ± 122	765 ± 97	1426 ± 241	823 ± 92	NMFES+K ***p* = 0.008** K NS	NMFES+K ***p* = 0.009** K ***p* = 0.009**	NMFES+K NS K ***p* = 0.009**
-anterior tibial muscle	1625 ± 324	708 ± 125	821 ± 192	1325 ± 96	728 ± 77	1321 ± 102	894 ± 126	NMFES+K ***p* = 0.009** K NS	NMFES+K ***p *= 0.008** K ***p* = 0.009**	NMFES+K NS K ***p* = 0.008**
-calf muscles	1621 ± 225	504 ± 128	525 ± 171	628 ± 245	522 ± 175	1407 ± 205	525 ± 582	NMFES+K ***p* = 0.008** K NS	NMFES+K ***p* = 0.009** K ***p* = 0.009**	NMFES+K NS K ***p* = 0.009**
sEMG (Index of Antagonistic Muscle Alternate Action)										
-at wrist	5	3	3	5	3	5	3	NMFES+K ***p* = 0.03** K NS	NMFES+K ***p* =0.03** K ***p* = 0.03**	NMFES+K NS K ***p* = 0.03**
-at ankle	5	3	3	4	3	4	3	NMFES+K ***p* = 0.05** K NS	NMFES+K ***p* = 0.03** K ***p* = 0.03**	NMFES+K ***p* = 0.04** K ***p* = 0.03**

Index of antagonistic muscle alternate action: 5: full alternate contraction; 4: slightly disturbed alternate contraction; 3: moderate disturbed alternate contraction; 2: highly disturbed alternate contraction; 1: no alternate contraction; rEMG: electromyographic recordings at rest; mcEMG: electromyographic recordings during maximal contraction lasting 5 s.

**Table 4:** Spearman’s rank correlation (r_s_) of tests results obtained on the symptomatic side before and after treatment in the two groups of patients. Cumulative data from the wrist flexors and calf muscles are presented. *p* ≤ 0.05 was assumed as statistically significant for rank correlation.

	NMFES+K Group, N = 60				K Group, N = 60			
	Before Treatment (T0)		After Treatment (T2)		Before Treatment (T0)		After Observation (T2)	
Parameter	Ashworth’s Scale (+4–1)							
rEMG	r_s_	*p*	r_s_	*p*	r_s_	*p*	r_s_	*p*
	0.725	0.003	0.652	0.002	0.722	0.003	0.699	0.001
Lovett’s Scale (0–5)								
mcEMG	r_s_	*p*	r_s_	*p*	r_s_	*p*	r_s_	*p*
	0.745	0.002	0.711	0.002	0.771	0.002	0.621	0.001
				rEMG				
mcEMG	r_s_	*p*	r_s_	*p*	r_s_	*p*	r_s_	*p*
	−0.689	0.003	−0.653	0.002	−0.655	0.003	−0.611	0.001

Abbreviations: rEMG: resting EMG amplitude; mcEMC: maximal contraction EMG amplitude.

## Data Availability

All data generated or analyzed during this study are included in this published article.
